# Myo-granules Connect Physiology and Pathophysiology

**DOI:** 10.1177/1179069519842157

**Published:** 2019-04-12

**Authors:** Alicia A Cutler, Theodore Eugene Ewachiw, Giulia A Corbet, Roy Parker, Brad B Olwin

**Affiliations:** 1 Department of Molecular, Cellular & Developmental Biology, University of Colorado Boulder, Boulder, CO, USA; 2 Department of Biochemistry, University of Colorado Boulder, Boulder, CO, USA

**Keywords:** Amyotrophic lateral sclerosis, murine

## Abstract

A hallmark of many neuromuscular diseases including Alzheimer disease, inclusion body myositis, amyotrophic lateral sclerosis, frontotemporal lobar dementia, and ocular pharyngeal muscular dystrophy is large cytoplasmic aggregates containing the RNA-binding protein, TDP-43. Despite acceptance that cytoplasmic TDP-43 aggregation is pathological, cytoplasmic TDP-43 assemblies form in healthy regenerating muscle. These recently discovered ribonucleoprotein assemblies, termed myo-granules, form in healthy muscle following injury and are readily cleared as the myofibers mature. The formation and dissolution of myo-granules during normal muscle regeneration suggests that these amyloid-like oligomers may be functional and that perturbations in myo-granule kinetics or composition may promote pathological aggregation.

**COMMENT ON:** Vogler TO, Wheeler JR, Nguyen ED, et al. TDP-43 and RNA form amyloid-like myo-granules in regenerating muscle. *Nature*. 2018;563:508-513. doi:10.1038/s41586-018-0665-2. PubMed PMID:30464263 https://www.ncbi.nlm.nih.gov/pubmed/30464263.

The connection between cytoplasmic protein aggregates and degenerative neuromuscular diseases has long fueled assumptions that these aggregates are inherently toxic and are primary drivers of pathogenesis. In disease, pathological aggregates accumulate and persist in affected cells. However, we discovered that amyloid like, cytoplasmic, ribonucleoprotein (RNP) assemblies form and are cleared during differentiation and regeneration of healthy skeletal muscle cells. This unexpected finding suggests that protein assemblies typically associated with disease are not toxic, but, in fact, may be beneficial and may perform a critical, non-pathological role during skeletal muscle cell maturation.

The RNA-binding protein TDP-43 is essential for skeletal muscle regeneration as deletion of a single allele of the *Tardbp* gene impairs muscle regeneration.^1^ In mature muscle, as in most cell types, TDP-43 is primarily nuclear and contributes to transcriptional regulation, splicing, and RNA stability.^2^ However, following muscle injury, cytosolic TDP-43 transiently increases forming higher order, amyloid-like assemblies called myo-granules. With sizes ranging from 50 to 250 nm, myo-granules are large and enriched for RNA-binding proteins and mRNAs encoding sarcomeric structural proteins.^1^ Unlike pathological aggregates, which persist in cells, myo-granules are cleared from differentiating muscle cells within 10 days following muscle injury,^1^ demonstrating that myofibers effectively clear these amyloid-like oligomeric assemblies.

Exciting possibilities arise from the discovery that myo-granules form and are cleared in healthy muscle. The existence of myo-granules, a previously unknown feature in skeletal muscle formation, disputes the assumption that amyloid-like oligomers are inherently pathological, and thus, myo-granules critically connect physiology and pathophysiology. Clarifying myo-granule biology will increase our understanding of skeletal muscle regeneration, neuromuscular diseases, and neuronal degenerative diseases with large cytoplasmic protein aggregates.

Myo-granules may help orchestrate sarcomere formation and organization during muscle formation. Sarcomeric proteins, which make up more than 60% of myofiber protein content,^3^ must be produced and organized to establish functional sarcomeres, the contractile unit of skeletal and cardiac muscle. Aberrations in sarcomere composition or organization impair muscle function.^4^ Transcripts encoding sarcomeric proteins are extremely long. While the average human mRNA is 3.3 kilobases (kb) long,^5^ myosin heavy chain transcripts are twice that^6^ and other sarcomeric transcripts are far larger: nebulin transcripts are approximately 25 kb,^7^ and the massive titin mRNA is more than 100 kb.^8^ Localized translation used by neurons and myofibers^9,10^ may permit translation of sarcomeric proteins at growing sarcomeres. The logistical challenge of packaging and transporting these large mRNAs to the appropriate subcellular locations may be solved by myo-granules (Figure 1). Myo-granules contain mRNA-binding proteins, proteins that repress translation, and many of the large mRNAs encoding sarcomeric proteins.^1^ Moreover, myo-granules surround sites of newly forming sarcomeres during muscle regeneration, and thus, myo-granules may transport and repress sarcomeric mRNA translation serving a similar role as neuronal messenger ribonucleoprotein (mRNP) transport granules.^11^

**Figure 1.**
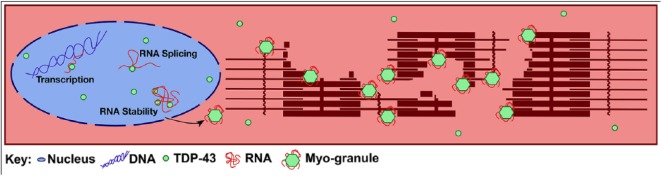
TDP-43 and myo-granule functions in myofibers. In skeletal muscle, TDP-43 regulates transcription, participates in RNA splicing, and promotes mRNA stability. In addition, we suggest that, as a myo-granule component, TDP-43 is involved in mRNA transport to locations of sarcomere formation.

Muscle biopsies from patients with inclusion body myositis (IBM),^12^ oculopharyngeal muscular dystrophy (OPMD),^13^ amyotrophic lateral sclerosis (ALS),^14^ and multisystem proteinopathy^15^ contain large TDP-43-containing cytoplasmic protein aggregates. A comparison of myo-granule composition in normal muscle formation with aggregates in diseases will determine whether normal myo-granules are related to disease-associated aggregates. Because isolated myo-granules share structural characteristics with disease-associated amyloid oligomers and spontaneously assemble into large amyloids,^1^ myo-granules may seed aggregates found in diseases through a combination of increased myo-graule generation and decreased myo-granule clrearance (Figure 2).

**Figure 2.**
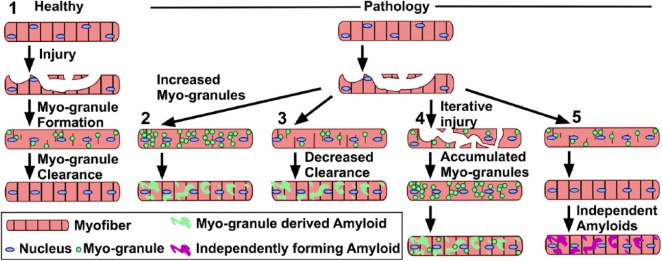
Myo-granules in healthy muscle and pathological amyloid formation: (1) in healthy muscle, myo-granules form following injury and are cleared as the myofiber matures. Large amyloid aggregates form in pathology and could result from (2) increased myo-granules production, (3) decreased myo-granule clearance, (4) increased production and decreased clearance as the result of iterative injury, and (5) the pathological amyloids may be unrelated to myo-granules and myo-granules may form and be cleared normally in pathology.

One potential mechanism to account for aggregate accumulation in myopathies is that they accumulate as a consequence of the iterative rounds of degeneration and regeneration occurring to repair muscle in degenerative muscle diseases. The asynchronous regeneration occurring in diseased muscle interferes with the immune response, resulting in a proinflammatory positive feedback loop that amplifies disease-associated fibrosis.^16^ Because myo-granules form in regenerating myofibers, ongoing regeneration could perturb myo-granule clearance, increasing myo-granules concentration, which promotes nucleation into larger, pathological aggregates.

Cytoplasmic protein aggregates are observed in many diseased organs including skeletal muscle, kidney, brain, heart, and the eye. In some cases, similar genetic mutations manifest in different organs in different patients,^17^ indicating that common mechanisms may be responsible for disease progression in different organs. Cytoplasmic TDP-43 aggregates are not exclusive to muscle formation, neurodegenerative diseases, or progressive neuromuscular diseases.^12,18-20^ TDP-43 transiently relocalizes to the cytoplasm of neurons following traumatic brain injury,^21^ hinting that amyloid-like oligomers may have a similar roles in neuronal and skeletal muscle recovery. Although we do not know the extent to which myo-granules and neuronal aggregates are analogous, similar mechanisms may govern their formation and clearance. Understanding how clearance pathways operate in skeletal muscle may lead to therapies aimed at preventing or clearing pathological aggregates in muscle and other tissues.

The discovery of myo-granules as a previously unrecognized feature of skeletal muscle formation provides new knowledge to develop tools for deepening our understanding of skeletal muscle formation and challenges the accepted view that amyloid assemblies are inherently pathological. Examining myo-granule formation, composition, and clearance will clarify whether myo-granules directly contribute to large aggregate accumulation in neuromuscular diseases. Finally, elucidating the processes regulating myo-granules and amyloid aggregates in muscle may enhance our understanding of neuronal aggregates and provide translational applications for neuromuscular diseases.
